# Sphingolipid Metabolism and Transport in *Chlamydia trachomatis* and *Chlamydia psittaci* Infections

**DOI:** 10.3389/fcell.2019.00223

**Published:** 2019-10-04

**Authors:** Sebastian Banhart, Elena K. Schäfer, Jean-Marc Gensch, Dagmar Heuer

**Affiliations:** Unit ‘Sexually Transmitted Bacterial Infections’, Department for Infectious Diseases, Robert Koch Institute, Berlin, Germany

**Keywords:** *Chlamydia*, sphingolipid, sphingomyelin (SM), ceramide (CER), CERT (CERamide Transfer protein), Inc proteins, infection, RAB proteins

## Abstract

*Chlamydia* species infect a large range of vertebral hosts and have become of major economic and public health concern over the last decades. They are obligate intracellular bacteria that undergo a unique cycle of development characterized by the presence of two distinct bacterial forms. After infection of the host cell, *Chlamydia* are found inside a membrane-bound compartment, the inclusion. The surrounding membrane of the inclusion contributes to the host-*Chlamydia* interface and specific pathogen-derived Inc proteins shape this interface allowing interactions with distinct cellular proteins. In contrast to many other bacteria, *Chlamydia* species acquire sphingomyelin from the host cell. In recent years a clearer picture of how *Chlamydia trachomatis* acquires this lipid emerged showing that the bacteria interact with vesicular and non-vesicular transport pathways that involve the recruitment of specific RAB proteins and the lipid-transfer protein CERT. These interactions contribute to the development of a new sphingomyelin-producing compartment inside the host cell. Interestingly, recruitment of CERT is conserved among different *Chlamydia* species including *Chlamydia psittaci*. Here we discuss our current understanding on the molecular mechanisms used by *C. trachomatis* and *C. psittaci* to establish these interactions and to create a novel sphingomyelin-producing compartment inside the host cell important for the infection.

## Introduction

Lipids are important factors in bacterial infections. They serve as energy source, structural components and are involved in the immune response. Like many bacteria, *Chlamydia trachomatis* is able to synthesize most phospholipids except for sphingomyelin, cholesterol and phosphatidylcholine. Sphingomyelin is mainly produced by eukaryotic cells thus; the detection of sphingomyelin inside chlamydial cells was astonishing. This review summarizes recent advances in our understanding of how *Chlamydia* spp. acquire sphingolipids from the host cell and describes their functions for *Chlamydia* biology.

## Clinics of *Chlamydia trachomatis* and *C. psittaci* Infections

*Chlamydia trachomatis* strains can be divided into biovars. The trachoma biovar (serovars A-C) can cause trachoma, the leading cause of preventable blindness that is hyperendemic in many rural areas of Africa, Central and South America, Asia, Australia and the Middle East. Infections with the urogenital tract biovar (serovars D-K) are among the most frequently sexually transmitted bacterial infections world-wide. They affect mainly young adults and persons with multiple sex partners (Newman et al., 2015). Symptoms range from asymptomatic to urethritis and proctitis in both genders and cervicitis in females. In particular, untreated or re-occurring infections in women have been associated with severe outcomes including pelvic inflammatory diseases (PID), ectopic pregnancies and infertility. Furthermore, during pregnancy, untreated *C. trachomatis* infections are a risk factor of preterm birth, conjunctivitis and pneumonia of the newborn. *C. trachomatis* belonging to the lymphogranuloma venereum biovar (LGV, L1-L3) is also sexually transmitted and can cause urogenital or anorectal infections in humans that can be more invasive by disseminating to the lymph nodes (Elwell et al., 2016).

*Chlamydia psittaci* is a zoonotic pathogen that causes respiratory disease in humans and avian species, also known as psittacosis or ornithosis (Knittler et al., 2014; Knittler and Sachse, 2015). The agent was originally isolated from birds, but meanwhile it has been found in different mammalian hosts like cattle, horses and pigs (Longbottom and Coulter, 2003). *C. psittaci* can be transmitted from domestic birds to humans by inhalation of aerosolized bacteria from the feces of infected avian species (Knittler et al., 2014; Knittler and Sachse, 2015). In many cases *C. psittaci* infections remain undetected and undiagnosed due to unspecific symptoms (fever, chills, headache, malaise, myalgia) (Knittler et al., 2014; Knittler and Sachse, 2015).

## Biology of *Chlamydiacea*e

Both *C. trachomatis* and *C. psittaci* belong to the family of *Chlamydiaceae*. A hallmark of all members of this family is their obligate intracellular, biphasic cycle of development that takes place in a membrane-bound compartment inside a eukaryotic host cell (Moulder, 1991; Hybiske, 2015).

**FIGURE 1:**
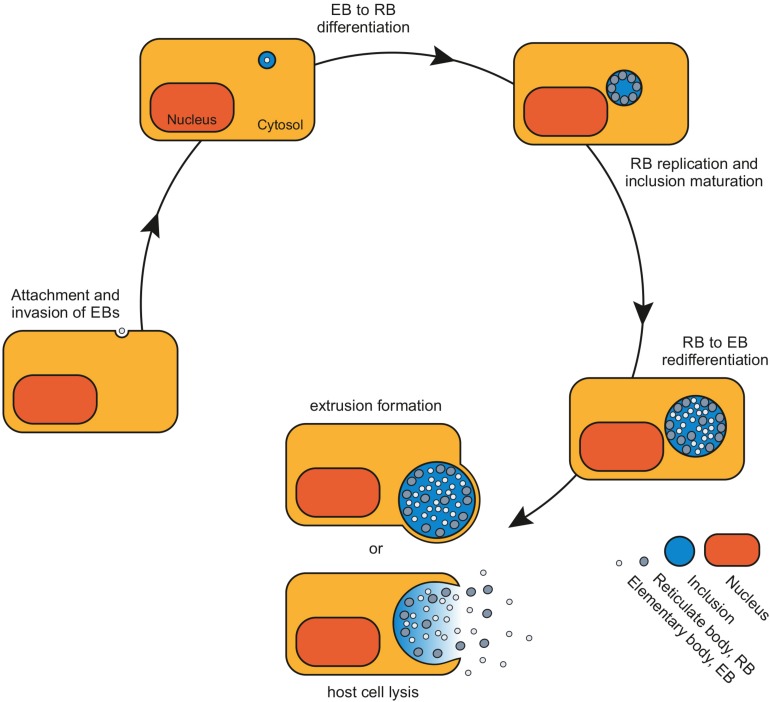
The chlamydial developmental cycle. The biphasic developmental cycle of *Chlamydia* spp. starts with the attachment and invasion of host cells by infectious elementary bodies (EBs). Within their membrane-bound vacuole, termed inclusion, EBs differentiate into metabolically active reticulate bodies (RBs). RBs undergo repeated cycles of replication before they finally re-differentiate into EBs. The life cycle ends with the release of EBs from the host cell by either host cell lysis or extrusion formation to start a new round of infection.

It is characterized by the switch between the extracellular, infectious elementary bodies (EBs) and the intracellular, non-infectious, metabolically active reticulate bodies (RBs) (Figure 1). EBs are 0.3 μm in size and enter the host cell by receptor-mediated endocytosis or phagocytosis, involving bacterial adhesins, host cell receptors, and host-specific heparan proteoglycans (Elwell et al., 2016). After internalization, EBs are found in vacuoles, termed inclusions that protect the bacteria from the immune response of the host cell. By releasing effector molecules into the host cell via a type III secretion system, the inclusion membrane is modified and can escape the phagolysosomal pathway (Moore and Ouellette, 2014). Within the inclusion, EBs differentiate into the osmotically instable RBs. These 1 μm small, structurally flexible bacteria divide asymmetrically (Nunes and Gomes, 2014; Abdelrahman et al., 2016). RBs synthesize a family of special proteins, the Inc proteins, which are unique to *Chlamydia* spp. and are integral parts of the bacterial inclusion membrane. They are important bacterial constituents of the inclusion-host cell interface and confer stability to the inclusion membrane (Mirrashidi et al., 2015; Weber et al., 2017). Inc proteins were originally identified in *C. psittaci*. They are a family of *Chlamydia*-specific proteins lacking sequence homology to any known proteins, or to themselves. Interestingly, genomic comparison of different *Chlamydia* strains showed that some Inc proteins are conserved between different species and others are species-specific. These non-conserved Inc proteins may be involved in tissue tropism (Dehoux et al., 2011; Lutter et al., 2012).

At 16–20 h *post infection* (*p.i.*) some RBs start to transform back into EBs, while other RBs continue to replicate. Depending on chlamydial species and growth conditions, at 48–72 h *p.i.* both developmental stages are released from the host cell by either complete lysis of the host cell or by a mechanism called extrusion – the release of the intact inclusion enveloped by host cell plasma membrane (Figure 1; Hybiske and Stephens, 2007). Freed EBs can infect neighboring host cells and start a new round of infection.

## Sphingolipid Synthesis in Eukaryotic Cells

Sphingolipids are major integral components of eukaryotic cell membranes. They function as structural and signaling molecules that can regulate apoptosis, cellular proliferation and stress responses (Heung et al., 2006; Breslow and Weissman, 2010). Defects in sphingolipid metabolism have been linked to different diseases including carcinogenesis, cardiovascular and neurodegenerative diseases (Heung et al., 2006).

A sphingoid base linked to a specific fatty acid is the building block of the diverse family of sphingolipids. In sphingomyelin, this backbone is linked to a head group of phosphocholine whereas complex glycosphingolipids are generated by addition of a specific sugar residue. Sphingolipids are a family of structurally and functionally diverse lipids and are synthesized by three distinct pathways: (1) *de novo* synthesis, (2) sphingomyelinase pathways, and (3) salvage pathway that involve specific enzymes localized to distinct organelles inside the cell.

*De novo* sphingolipid synthesis begins with condensation of serine and palmitoyl coenzyme A (CoA) which takes place at the cytosolic leaflet of the endoplasmic reticulum (ER) catalyzed by the highly conserved palmitoyltransferase (SPTLC) (Yard et al., 2007; Breslow and Weissman, 2010; Hannun and Obeid, 2018). The product of SPTLC, 3-ketosphinganine, is further reduced by 3-ketosphinganine reductase (KDSR) and N-acylated by the action of fatty acid specific dihydroceramide synthases (CERS1-6). Finally, dihydroceramide is desaturated by dihydroceramide desaturase (DEGS) to generate ceramide. Ceramide represents the central precursor molecule of the sphingolipid metabolism, which in turn is used to generate several sphingolipids, like sphingomyelin, sphingosine or complex glycosphingolipids. Ceramide is then transported from the ER to the Golgi apparatus by vesicular trafficking or by transport proteins (Bartke and Hannun, 2009; Hanada, 2010). Within the Golgi, ceramides are modified at the head-group position by adding phosphocholine and phosphate to produce sphingomyelin and ceramide 1-phosphate (Tafesse et al., 2006; Hannun and Obeid, 2018). Ceramide is converted to sphingomyelin by sphingomyelin synthases (SMS) located at the lumen of the *trans-Golgi* (SMS1 and SMS2) and at the plasma membrane (SMS2) (Tafesse et al., 2006; Yamaji and Hanada, 2015). Precursor of complex glycosphingolipids, such as glucosylceramide and galactosylceramide, are formed by the addition of glucose and galactose residues in a glycosidic linkage to ceramide (Breslow and Weissman, 2010; Yamaji and Hanada, 2015). Ultimately, sphingolipids and glycosphingolipids are transported through secretory pathways to plasma membranes and subcellular organelles.

Alternatively, ceramides can be generated by the breakdown of complex sphingolipids, termed salvage pathway (Kitatani et al., 2008). Sphingolipids and glycosphingolipids are degraded in acidic subcellular compartments, such as late endosomes and lysosomes, to form sphingosine (Kitatani et al., 2008). In contrast to ceramide, which is not capable to leave the lysosome, sphingosine is able to enter different cell compartments (Bartke and Hannun, 2009). Released sphingosine may re-enter sphingolipid pathways and is reused by the ceramide synthase to generate ceramides again via re-acylation (Kitatani et al., 2008).

The third pathway, termed sphingomyelinase pathway, occurs in the plasma membrane and endosome/lysosome systems (Yamaji and Hanada, 2015; Teo et al., 2016). Within these compartments, sphingomyelin is converted to ceramide by acid sphingomyelinases (Kitatani et al., 2008). At plasma membranes, SMS2 adds phosphocholine head groups to ceramide, which leads to the production of sphingomyelin.

## Sphingolipid Transport in *Chlamydia-*Infected Cells

Twenty four years ago, Hackstadt et al. (1995) showed that fluorescently labeled sphingomyelin is acquired by *C. trachomatis* from the host cell. Based on the observation that purified EBs contained fluorescent sphingomyelin the authors concluded that Golgi-derived sphingomyelin accumulates in bacteria rather than its precursor ceramide. Classical protein markers of the transport between the Golgi apparatus and the plasma membrane were not found in the inclusion membrane suggesting that a subset of Golgi-derived exocytic vesicles is targeted (Hackstadt et al., 1996; Scidmore et al., 1996a). *C. trachomatis* protein synthesis is required for this interaction and bacterial factors that mediate fusogenicity with sphingomyelin-containing vesicles seem to be continually replenished (Scidmore et al., 1996b, 2003). Shortly after these initial observations, quantitative analysis indicated that *C. trachomatis* membranes contain up to 4% of sphingolipids (Wylie et al., 1997). Interfering with bacterial sphingolipid acquisition resulted in less infectious bacteria, leads to the formation of aberrant chlamydial forms and demonstrated the requirement of sphingolipid metabolism for reactivation after INFγ treatment of *C. trachomatis*-infected cells (van Ooij et al., 2000; Rejman Lipinski et al., 2009; Robertson et al., 2009). Interestingly, synthesis of sphingomyelin from ceramide seems to be a prerequisite for sphingolipid uptake into the inclusion and into the bacteria, as a ceramide derivative that cannot be converted to sphingomyelin (1-*O*-methyl-ceramide) was not translocated across the inclusion membrane but rather accumulated around the inclusion (Banhart et al., 2014). This ceramide derivative showed strong anti-chlamydial activity suggesting that *C. trachomatis* generates a sphingomyelin-producing compartment inside the host cell which is important for chlamydial growth (Banhart et al., 2014; Saied et al., 2015). Surprisingly, a recent study suggests that sphingomyelin uptake by *Chlamydia* species is linked to host adaptation and/or virulence rather than to its obligate intracellular life style (Dille et al., 2015).

**FIGURE 2:**
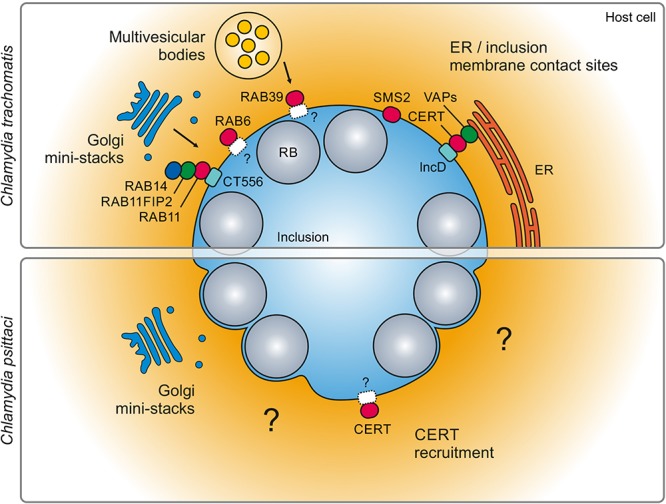
Sphingolipid acquisition during *Chlamydia* infection. Acquisition of sphingolipids takes place by both vesicular and non-vesicular pathways and is ensured by interactions with several subcellular compartments and host cell proteins. Vesicular transport of sphingolipids to *C. trachomatis* is realized by rerouting vesicles from fragmented Golgi mini-stacks or multivesicular bodies, involving several RAB GTPases such as RAB6, RAB11, RAB14, and RAB39. Recruitment of RAB GTPases is thought to be mediated by Inc proteins and interaction of RAB11 with CT556 has been described (Mirrashidi et al., 2015). Non-vesicular routes to *C. trachomatis* include recruitment of SMS and the formation of ER/inclusion membrane contact sites that contain the ceramide transport protein CERT. In contrast, little is known for *C. psittaci*, except for fragmentation of the Golgi apparatus and recruitment of CERT.

In recent years, vesicular and non-vesicular transport pathways were identified that were hijacked by *C. trachomatis* to obtain sphingolipids from the host cell (Figure 2). These pathways are not redundant and play distinct roles during the chlamydial cycle of development. It has been shown that *C. trachomatis* can intercept vesicular transport routes from different organelles including Golgi mini-stacks or multivesicular bodies (MVBs). Transport of sphingolipid-containing vesicles derived from Golgi mini-stacks requires cellular GTPases RAB14, RAB6A and RAB11A, ARF1 and its guanine nucleotide exchange factor GBF1 (Heuer et al., 2009; Elwell et al., 2011). Interestingly, RAB14, RAB6A, and RAB11A appear to be important for *Chlamydia* progeny formation whereas ARF1 and GBF1 seem to be dispensable (Rejman Lipinski et al., 2009; Capmany and Damiani, 2010; Elwell et al., 2011). The recruitment of RAB14-postive vesicles was shown to be controlled by the Akt signaling pathway, a pathway that is activated by *C. trachomatis* infections (Capmany et al., 2019). Several other kinases have also been implicated in sphingomyelin transport to the *C. trachomatis* inclusion. These include SRC family kinase Fyn and serine/threonine kinases that have been identified in an RNAi screen and based upon inhibition by rotterlin, respectively (Shivshankar et al., 2008; Mital and Hackstadt, 2011). The precise mechanisms how rotterlin inhibits sphingomyelin uptake by *C. trachomatis* remains elusive as rotterlin appears to have multiple targets inside the host cell (Lei et al., 2012). In addition, sphingomyelin is transported from MVBs by RAB39 (Gambarte Tudela et al., 2019). The MVB marker protein CD63 has been detected inside C. trachomatis inclusion but its functional role remains elusive (Beatty, 2006, 2008). In contrast, much less is known for sphingolipid acquisition in *C. psittaci* infections (Figure 2). For both species, infection results in fragmentation of the cellular Golgi apparatus into smaller Golgi mini-stacks thereby increasing Golgi surface (Heuer et al., 2009; Knittler et al., 2014). This phenotype has been shown to boost sphingolipid acquisition in *C. trachomatis* infections (Heuer et al., 2009). In sum, multiple cellular processes contribute to sphingolipid acquisition in *Chlamydia* infections (Moore, 2012). How these factors regulate sphingolipid transport and influence the infection is currently not completely understood.

The recruitment of cellular proteins, especially RAB proteins, is species dependent (Damiani et al., 2014). In the past, the localizations of RAB proteins were investigated during infection of different *Chlamydia* species and showed that a core subset of RAB proteins is recruited to the inclusion membrane of different *Chlamydia* species whereas a few RAB proteins are species-specific (Rzomp et al., 2003). Interestingly, although recruitment of a RAB protein is conserved between different *Chlamydia* species (Rab4 in *C. trachomatis* serovar L2 and D, *C. muridarum*, and *C. pneumoniae*), its identified bacterial interaction partner that is responsible for the interaction (CT229 in *C. trachomatis* serovar L2) has not been found in the other chlamydial species (Rzomp et al., 2006). This leaves a question mark on of how the mechanisms of functional recruitment differ between *Chlamydia* species.

Future research regarding the role of these different vesicular pathways in infections with different *Chlamydia* species, the identification of transported lipids and bacterial factors controlling these interactions is needed to understand the intricate relationship.

## Cert-Dependent Acquisition of Sphingolipids and Beyond

New studies showed that *C. trachomatis* and *C. psittaci* hijack the cellular ceramide transport protein CERT to obtain ceramide from the host cell (Derre et al., 2011; Koch-Edelmann et al., 2017). CERT transfers ceramide from the ER to the Golgi apparatus in uninfected cells using the C-terminal START domain (Ponting and Aravind, 1999). Its N-terminal pleckstrin homology (PH) domain binds phosphatidylinositol-4-phosphate (PI4P) (Peretti et al., 2008) at the *cis*-face of the Golgi apparatus and is linked with the ER due to its central FFAT motif binding VAPs (Vesicle-associated membrane protein-associated protein) (Loewen et al., 2003). In *C. trachomatis*-infected cells, CERT is recruited to the inclusion membrane by interaction with IncD (Figure 2). Targeted deletion of CERT domains showed that the FFAT motif is relevant for binding and co-recruiting VAPs to the inclusion membrane, but lack of the PH domain interrupts association to the inclusion (Agaisse and Derre, 2014). The interaction of IncD with CERT is driven by the charged and hydrophobic motif in its C-terminus as well as the charged motif in the N-terminus (Kumagai et al., 2018). These motifs are conserved in *C. trachomatis*, *C. suis*, *C. muridarum*, *C. caviae*, and *C. felis*. Also, the proximity of both domains and the possibility of forming homooligomers mediated by the transmembrane domain are necessary for increasing the affinity to CERT. After CERT recruitment to the inclusion membrane, ceramide is likely transported from the ER to the inclusion membrane at ER-inclusion contact sides where ceramide is subsequently converted into sphingomyelin by the also recruited host SMS2 (Figure 2; Elwell et al., 2011). IncD belongs to the non-conserved Inc proteins that are not found in all *Chlamydia* species, for example *C. psittaci*. Thus, it is currently not known how CERT is recruited to *C. psittaci* inclusions (Koch-Edelmann et al., 2017). In uninfected cells, the CERT PH domain binds to PI4P-enriched membranes in the *trans-Golgi* region. It has been suggested, that PI4P is present at *C. trachomatis* inclusion membranes and might thereby partially mediate CERT binding (Moorhead et al., 2010). Assuming that *C. psittaci* inclusions are PI4P positive, this mode of binding could be conserved between the different *Chlamydia* species. In addition, proteomic analysis of *C. trachomatis* inclusions revealed that VAPB, a binding partner of CERT is significantly enriched in the inclusion proteome (Aeberhard et al., 2015). Whether VAPB is also associated with *C. psittaci* inclusions or if a currently unknown *C. psittaci* factor facilitates CERT recruitment still needs to be determined. Thus, future experiments are needed to reveal the nature of CERT binding to *C. psittaci* inclusions.

Sphingomyelin is one of the essential host-derived lipids that is incorporated into chlamydial membranes (Saka and Valdivia, 2010) and is described to play a role in bacterial replication and inclusion growth (Hackstadt et al., 1996; Rejman Lipinski et al., 2009; Elwell et al., 2011). Further evidence for this suggestion is that CERT recruitment is conserved among *Chlamydia* spp. (Koch-Edelmann et al., 2017). For *C. trachomatis* and *C. muridarum* it has been shown by RNA interference that CERT seems to be essential for the production of infectious progeny, indicating that CERT is a crucial factor in chlamydial development (Derre et al., 2011; Elwell et al., 2011). Recent studies using CRISPR/Cas9-mediated CERT-knockout cells demonstrated that deficiency of CERT in *C. psittaci* infections also leads to decreased infectious progeny formation (Koch-Edelmann et al., 2017). Interestingly, CERT-knockout caused an increase of sphingolipid uptake by *C. psittaci* (Koch-Edelmann et al., 2017). This is in stark contrast to *C. trachomatis* infection that shows a drastic decrease in bacterial sphingolipid acquisition under CERT depletion. These findings possibly suggest a CERT-independent sphingolipid uptake pathway in *C. psittaci* infections. How sphingolipids are transported to *C. psittaci* in CERT-knockout cells is currently not known. The involvement of one or more novel factor/s of either bacterial and/or cellular origin that compensate for loss of CERT is likely. Besides that, these results underline that acquisition of sphingomyelin needs to be controlled by *Chlamydia* spp. and suggest that CERT might have additional roles in chlamydial development beyond sphingolipid transport, which need to be investigated in the future.

## Summary and Outlook

Twenty four years after the initial observation that *C. trachomatis* can acquire sphingomyelin from the Golgi apparatus of the infected host cells a clearer picture is emerging on the molecular pathways used by different *Chlamydia* species to obtain sphingolipids. *Chlamydia* species use distinct, non-redundant pathways to obtain sphingolipids. These include vesicular and non-vesicular transport pathways. The characterization of CERT as a conserved factor in ceramide delivery to different *Chlamydia* species and the recruitment of the human SMS2 to the *C. trachomatis* inclusion suggests that at least *C. trachomatis* creates a novel sphingomyelin-producing compartment inside the infected host cells. Additionally, in *C. trachomatis* infections sphingomyelin is transported by distinct vesicles. For that purpose, *C. trachomatis* exploits cellular GTPases, including RAB and ARF proteins, and kinases to facilitate bacterial sphingomyelin acquisition from fragmented Golgi mini-stacks and MVBs. How vesicular and non-vesicular transport of sphingolipids is controlled by different *Chlamydia* species, how they process CERT-delivered ceramide, and how ceramide and sphingomyelin regulate chlamydial infections are just a few open questions. The development of novel tools including the genetic manipulation of *Chlamydia* species and the biochemical isolation of chlamydial inclusions now allows addressing these questions.

## Author Contributions

DH developed the conception of the manuscript and wrote the manuscript. SB designed the figures. ES, J-MG, and SB contributed to and wrote sections of the manuscript. All authors contributed to the manuscript revision, read, and approved the submitted version of the manuscript.

## Conflict of Interest

The authors declare that the research was conducted in the absence of any commercial or financial relationships that could be construed as a potential conflict of interest.

## References

[B1] Abdelrahman Y. Ouellette S. P. Belland R. J. Cox J. V. (2016). Polarized cell division of *Chlamydia trachomatis*. *PLoS Pathog.* 12:e1005822. 10.1371/journal.ppat.1005822 27505160 PMC4978491

[B2] Aeberhard L. Banhart S. Fischer M. Jehmlich N. Rose L. Koch S. (2015). The proteome of the isolated *Chlamydia trachomatis* containing vacuole reveals a complex trafficking platform enriched for retromer components. *PLoS Pathog.* 11:e1004883. 10.1371/journal.ppat.1004883 26042774 PMC4456400

[B3] Agaisse H. Derre I. (2014). Expression of the effector protein IncD in *Chlamydia trachomatis* mediates recruitment of the lipid transfer protein CERT and the endoplasmic reticulum-resident protein VAPB to the inclusion membrane. *Infect. Immun.* 82 2037–2047. 10.1128/IAI.01530-14 24595143 PMC3993449

[B4] Banhart S. Saied E. M. Martini A. Koch S. Aeberhard L. Madela K. (2014). Improved plaque assay identifies a novel anti-Chlamydia ceramide derivative with altered intracellular localization. *Antimicrob. Agents Chemother.* 58 5537–5546. 10.1128/AAC.03457-14 25001308 PMC4135853

[B5] Bartke N. Hannun Y. A. (2009). Bioactive sphingolipids: metabolism and function. *J. Lipid Res.* 50(Suppl.), S91–S96. 19017611 10.1194/jlr.R800080-JLR200 PMC2674734

[B6] Beatty W. L. (2006). Trafficking from CD63-positive late endocytic multivesicular bodies is essential for intracellular development of *Chlamydia trachomatis*. *J. Cell Sci.* 119 350–359. 10.1242/jcs.02733 16410552

[B7] Beatty W. L. (2008). Late endocytic multivesicular bodies intersect the chlamydial inclusion in the absence of CD63. *Infect. Immun.* 76 2872–2881. 10.1128/IAI.00129-08 18426873 PMC2446703

[B8] Breslow D. K. Weissman J. S. (2010). Membranes in balance: mechanisms of sphingolipid homeostasis. *Mol. Cell* 40 267–279. 10.1016/j.molcel.2010.10.005 20965421 PMC2987644

[B9] Capmany A. Damiani M. T. (2010). Chlamydia trachomatis intercepts Golgi-derived sphingolipids through a Rab14-mediated transport required for bacterial development and replication. *PLoS One* 5:e14084. 10.1371/journal.pone.0014084 21124879 PMC2989924

[B10] Capmany A. Gambarte Tudela J. Alonso Bivou M. Damiani M. T. (2019). Akt/AS160 signaling pathway inhibition impairs infection by decreasing Rab14-controlled sphingolipids delivery to Chlamydial Inclusions. *Front. Microbiol.* 10:666. 10.3389/fmicb.2019.00666 31001235 PMC6456686

[B11] Damiani M. T. Gambarte Tudela J. Capmany A. (2014). Targeting eukaryotic Rab proteins: a smart strategy for chlamydial survival and replication. *Cell. Microbiol.* 16 1329–1338. 10.1111/cmi.12325 24948448

[B12] Dehoux P. Flores R. Dauga C. Zhong G. Subtil A. (2011). Multi-genome identification and characterization of chlamydiae-specific type III secretion substrates: the Inc proteins. *BMC Genomics* 12:109. 10.1186/1471-2164-12-109 21324157 PMC3048545

[B13] Derre I. Swiss R. Agaisse H. (2011). The lipid transfer protein CERT interacts with the Chlamydia inclusion protein IncD and participates to ER-Chlamydia inclusion membrane contact sites. *PLoS Pathog.* 7:e1002092. 10.1371/journal.ppat.1002092 21731489 PMC3121800

[B14] Dille S. Kleinschnitz E. M. Kontchou C. W. Nolke T. Hacker G. (2015). In contrast to *Chlamydia trachomatis*, *Waddlia chondrophila* grows in human cells without inhibiting apoptosis, fragmenting the Golgi apparatus, or diverting post-Golgi sphingomyelin transport. *Infect. Immun.* 83 3268–3280. 10.1128/IAI.00322-15 26056386 PMC4496594

[B15] Elwell C. Mirrashidi K. Engel J. (2016). Chlamydia cell biology and pathogenesis. *Nat. Rev. Microbiol.* 14 385–400. 10.1038/nrmicro.2016.30 27108705 PMC4886739

[B16] Elwell C. A. Jiang S. Kim J. H. Lee A. Wittmann T. Hanada K. (2011). *Chlamydia trachomatis* co-opts GBF1 and CERT to acquire host sphingomyelin for distinct roles during intracellular development. *PLoS Pathog.* 7:e1002198. 10.1371/journal.ppat.1002198 21909260 PMC3164637

[B17] Gambarte Tudela J. Buonfigli J. Lujan A. Alonso Bivou M. Cebrian I. Capmany A. (2019). Rab39a and Rab39b display different intracellular distribution and function in sphingolipids and phospholipids transport. *Int. J. Mol. Sci.* 20:E1688. 10.3390/ijms20071688 30987349 PMC6480249

[B18] Hackstadt T. Rockey D. D. Heinzen R. A. Scidmore M. A. (1996). *Chlamydia trachomatis* interrupts an exocytic pathway to acquire endogenously synthesized sphingomyelin in transit from the Golgi apparatus to the plasma membrane. *EMBO J.* 15 964–977. 10.1002/j.1460-2075.1996.tb00433.x 8605892 PMC449991

[B19] Hackstadt T. Scidmore M. A. Rockey D. D. (1995). Lipid metabolism in *Chlamydia trachomatis*-infected cells: directed trafficking of Golgi-derived sphingolipids to the chlamydial inclusion. *Proc Natl Acad Sci U.S.A.* 92 4877–4881. 10.1073/pnas.92.11.4877 7761416 PMC41810

[B20] Hanada K. (2010). Intracellular trafficking of ceramide by ceramide transfer protein. *Proc. Jpn. Acad. Ser. B Phys. Biol. Sci.* 86 426–437. 10.2183/pjab.86.426 20431265 PMC3417804

[B21] Hannun Y. A. Obeid L. M. (2018). Sphingolipids and their metabolism in physiology and disease. *Nat. Rev. Mol. Cell Biol.* 19 175–191. 10.1038/nrm.2017.107 29165427 PMC5902181

[B22] Heuer D. Rejman Lipinski A. Machuy N. Karlas A. Wehrens A. Siedler F. (2009). Chlamydia causes fragmentation of the Golgi compartment to ensure reproduction. *Nature* 457 731–735. 10.1038/nature07578 19060882

[B23] Heung L. J. Luberto C. Del Poeta M. (2006). Role of sphingolipids in microbial pathogenesis. *Infect. Immun.* 74 28–39. 10.1128/iai.74.1.28-39.2006 16368954 PMC1346627

[B24] Hybiske K. (2015). Expanding the molecular toolkit for Chlamydia. *Cell Host Microbe* 18 11–13. 10.1016/j.chom.2015.06.016 26159716

[B25] Hybiske K. Stephens R. S. (2007). Mechanisms of host cell exit by the intracellular bacterium Chlamydia. *Proc. Natl. Acad. Sci. U.S.A.* 104 11430–11435. 10.1073/pnas.0703218104 17592133 PMC2040915

[B26] Kitatani K. Idkowiak-Baldys J. Hannun Y. A. (2008). The sphingolipid salvage pathway in ceramide metabolism and signaling. *Cell. Signal.* 20 1010–1018. 10.1016/j.cellsig.2007.12.006 18191382 PMC2422835

[B27] Knittler M. R. Berndt A. Bocker S. Dutow P. Hanel F. Heuer D. (2014). *Chlamydia psittaci*: new insights into genomic diversity, clinical pathology, host-pathogen interaction and anti-bacterial immunity. *Int. J. Med. Microbiol.* 304 877–893. 10.1016/j.ijmm.2014.06.010 25082204

[B28] Knittler M. R. Sachse K. (2015). *Chlamydia psittaci*: update on an underestimated zoonotic agent. *Pathog. Dis.* 73 1–15. 10.1093/femspd/ftu007 25853998

[B29] Koch-Edelmann S. Banhart S. Saied E. M. Rose L. Aeberhard L. Laue M. (2017). The cellular ceramide transport protein CERT promotes *Chlamydia psittaci* infection and controls bacterial sphingolipid uptake. *Cell. Microbiol.* 19:e12752. 10.1111/cmi.12752 28544656

[B30] Kumagai K. Elwell C. A. Ando S. Engel J. N. Hanada K. (2018). Both the N- and C- terminal regions of the Chlamydial inclusion protein D (IncD) are required for interaction with the pleckstrin homology domain of the ceramide transport protein CERT. *Biochem. Biophys. Res. Commun.* 505 1070–1076. 10.1016/j.bbrc.2018.09.168 30314703 PMC6219635

[B31] Lei L. Li Z. Zhong G. (2012). Rottlerin-mediated inhibition of *Chlamydia trachomatis* growth and uptake of sphingolipids is independent of p38-regulated/activated protein kinase (PRAK). *PLoS One* 7:e44733. 10.1371/journal.pone.0044733 22970301 PMC3436872

[B32] Loewen C. J. Roy A. Levine T. P. (2003). A conserved ER targeting motif in three families of lipid binding proteins and in Opi1p binds VAP. *EMBO J.* 22 2025–2035. 10.1093/emboj/cdg201 12727870 PMC156073

[B33] Longbottom D. Coulter L. J. (2003). Animal chlamydioses and zoonotic implications. *J. Comp. Pathol.* 128 217–244. 10.1053/jcpa.2002.0629 12834606

[B34] Lutter E. I. Martens C. Hackstadt T. (2012). Evolution and conservation of predicted inclusion membrane proteins in chlamydiae. *Comp. Funct. Genomics* 2012:362104. 10.1155/2012/362104 22454599 PMC3290821

[B35] Mirrashidi K. M. Elwell C. A. Verschueren E. Johnson J. R. Frando A. Von Dollen J. (2015). Global mapping of the Inc-human interactome reveals that retromer restricts chlamydia infection. *Cell Host Microbe* 18 109–121. 10.1016/j.chom.2015.06.004 26118995 PMC4540348

[B36] Mital J. Hackstadt T. (2011). Role for the SRC family kinase Fyn in sphingolipid acquisition by chlamydiae. *Infect. Immun.* 79 4559–4568. 10.1128/IAI.05692-11 21896774 PMC3257913

[B37] Moore E. R. (2012). Sphingolipid trafficking and purification in *Chlamydia trachomatis*-infected cells. *Curr. Protoc. Microbiol.* Chapter 11:2. 10.1002/9780471729259.mc11a02s27 PMC3536446 23184593

[B38] Moore E. R. Ouellette S. P. (2014). Reconceptualizing the chlamydial inclusion as a pathogen-specified parasitic organelle: an expanded role for Inc proteins. *Front. Cell. Infect. Microbiol.* 4:157. 10.3389/fcimb.2014.00157 25401095 PMC4215707

[B39] Moorhead A. M. Jung J. Y. Smirnov A. Kaufer S. Scidmore M. A. (2010). Multiple host proteins that function in phosphatidylinositol-4-phosphate metabolism are recruited to the chlamydial inclusion. *Infect. Immun.* 78 1990–2007. 10.1128/IAI.01340-09 20231409 PMC2863499

[B40] Moulder J. W. (1991). Interaction of chlamydiae and host cells in vitro. *Microbiol. Rev.* 55 143–190. 2030670 10.1128/mr.55.1.143-190.1991 PMC372804

[B41] Newman L. Rowley J. Vander Hoorn S. Wijesooriya N. S. Unemo M. Low N. (2015). Global estimates of the prevalence and incidence of four curable sexually transmitted infections in 2012 based on systematic review and global reporting. *PLoS One* 10:e0143304. 10.1371/journal.pone.0143304 26646541 PMC4672879

[B42] Nunes A. Gomes J. P. (2014). Evolution, phylogeny, and molecular epidemiology of Chlamydia. *Infect. Genet. Evol.* 23 49–64. 10.1016/j.meegid.2014.01.029 24509351

[B43] Peretti D. Dahan N. Shimoni E. Hirschberg K. Lev S. (2008). Coordinated lipid transfer between the endoplasmic reticulum and the Golgi complex requires the VAP proteins and is essential for Golgi-mediated transport. *Mol. Biol. Cell* 19 3871–3884. 10.1091/mbc.E08-05-0498 18614794 PMC2526681

[B44] Ponting C. P. Aravind L. (1999). START: a lipid-binding domain in StAR, HD-ZIP and signalling proteins. *Trends Biochem. Sci.* 24 130–132. 10.1016/s0968-0004(99)01362-6 10322415

[B45] Rejman Lipinski A. Heymann J. Meissner C. Karlas A. Brinkmann V. Meyer T. F. (2009). Rab6 and Rab11 regulate *Chlamydia trachomatis* development and golgin-84-dependent Golgi fragmentation. *PLoS Pathog.* 5:e1000615. 10.1371/journal.ppat.1000615 19816566 PMC2752117

[B46] Robertson D. K. Gu L. Rowe R. K. Beatty W. L. (2009). Inclusion biogenesis and reactivation of persistent *Chlamydia trachomatis* requires host cell sphingolipid biosynthesis. *PLoS Pathog.* 5:e1000664. 10.1371/journal.ppat.1000664 19936056 PMC2774160

[B47] Rzomp K. A. Moorhead A. R. Scidmore M. A. (2006). The GTPase Rab4 interacts with *Chlamydia trachomatis* inclusion membrane protein CT229. *Infect. Immun.* 74 5362–5373. 10.1128/iai.00539-06 16926431 PMC1594829

[B48] Rzomp K. A. Scholtes L. D. Briggs B. J. Whittaker G. R. Scidmore M. A. (2003). Rab GTPases are recruited to chlamydial inclusions in both a species-dependent and species-independent manner. *Infect. Immun.* 71 5855–5870. 10.1128/iai.71.10.5855-5870.2003 14500507 PMC201052

[B49] Saied E. M. Banhart S. Burkle S. E. Heuer D. Arenz C. (2015). A series of ceramide analogs modified at the 1-position with potent activity against the intracellular growth of *Chlamydia trachomatis*. *Future Med. Chem.* 7 1971–1980. 10.4155/fmc.15.126 26496536

[B50] Saka H. A. Valdivia R. H. (2010). Acquisition of nutrients by Chlamydiae: unique challenges of living in an intracellular compartment. *Curr. Opin. Microbiol.* 13 4–10. 10.1016/j.mib.2009.11.002 20006538 PMC3202608

[B51] Scidmore M. A. Fischer E. R. Hackstadt T. (1996a). Sphingolipids and glycoproteins are differentially trafficked to the *Chlamydia trachomatis* inclusion. *J. Cell Biol.* 134 363–374. 10.1083/jcb.134.2.363 8707822 PMC2120880

[B52] Scidmore M. A. Rockey D. D. Fischer E. R. Heinzen R. A. Hackstadt T. (1996b). Vesicular interactions of the *Chlamydia trachomatis* inclusion are determined by chlamydial early protein synthesis rather than route of entry. *Infect. Immun.* 64 5366–5372. 8945589 10.1128/iai.64.12.5366-5372.1996 PMC174531

[B53] Scidmore M. A. Fischer E. R. Hackstadt T. (2003). Restricted fusion of *Chlamydia trachomatis* vesicles with endocytic compartments during the initial stages of infection. *Infect. Immun.* 71 973–984. 10.1128/iai.71.2.973-984.2003 12540580 PMC145390

[B54] Shivshankar P. Lei L. Wang J. Zhong G. (2008). Rottlerin inhibits chlamydial intracellular growth and blocks chlamydial acquisition of sphingolipids from host cells. *Appl. Environ. Microbiol.* 74 1243–1249. 10.1128/aem.02151-07 18083882 PMC2258579

[B55] Tafesse F. G. Ternes P. Holthuis J. C. (2006). The multigenic sphingomyelin synthase family. *J. Biol. Chem.* 281 29421–29425. 10.1074/jbc.r600021200 16905542

[B56] Teo W. X. Kerr M. C. Huston W. M. Teasdale R. D. (2016). Sortilin is associated with the chlamydial inclusion and is modulated during infection. *Biol. Open* 5 429–435. 10.1242/bio.016485 26962046 PMC4890668

[B57] van Ooij C. Kalman L. Ijzendoorn van P. Nishijima M. Hanada K. Mostov K. (2000). Host cell-derived sphingolipids are required for the intracellular growth of *Chlamydia trachomatis*. *Cell. Microbiol.* 2 627–637. 10.1046/j.1462-5822.2000.00077.x 11207614

[B58] Weber M. M. Lam J. L. Dooley C. A. Noriea N. F. Hansen B. T. Hoyt F. H. (2017). Absence of specific *Chlamydia trachomatis* inclusion membrane proteins triggers premature inclusion membrane lysis and host cell death. *Cell Rep.* 19 1406–1417. 10.1016/j.celrep.2017.04.058 28514660 PMC5499683

[B59] Wylie J. L. Hatch G. M. McClarty G. (1997). Host cell phospholipids are trafficked to and then modified by *Chlamydia trachomatis*. *J. Bacteriol.* 179 7233–7242. 10.1128/jb.179.23.7233-7242.1997 9393685 PMC179671

[B60] Yamaji T. Hanada K. (2015). Sphingolipid metabolism and interorganellar transport: localization of sphingolipid enzymes and lipid transfer proteins. *Traffic* 16 101–122. 10.1111/tra.12239 25382749

[B61] Yard B. A. Carter L. G. Johnson K. A. Overton I. M. Dorward M. Liu H. (2007). The structure of serine palmitoyltransferase; gateway to sphingolipid biosynthesis. *J. Mol. Biol.* 370 870–886. 17559874 10.1016/j.jmb.2007.04.086

